# Radiative cooling: arising from practice and in turn serving practice

**DOI:** 10.1515/nanoph-2023-0678

**Published:** 2024-01-25

**Authors:** Quan Zhang, Zhonghao Rao, Rujun Ma

**Affiliations:** Hebei Engineering Research Center of Advanced Energy Storage Technology and Equipment, School of Energy and Environmental Engineering, Hebei University of Technology, Tianjin 300401, China; School of Materials Science and Engineering, National Institute for Advanced Materials, Nankai University, Tianjin 300350, China

**Keywords:** radiative cooling, thermal photonics, sustainable development, net zero-energy consumption, practicality

## Abstract

Radiative cooling, as a renewable cooling technology, is expected to mitigate growing global warming. However, the barrier when promoting radiative cooling from the laboratory to practice is still a blind spot and needs to be discussed right now. Here, on the basis of review for brief history, we propose a developing thread that the studies on radiative cooling arise from practice and in turn serves practice at the end. This perspective orderly elaborates fundamental limit in theory, realization of spectral-selective materials, practice on criteria for cooling performance, challenges and corresponding possible solutions in practice, and focusing on serving practice. We hope that the criticism for our own opinion could trigger researchers to deeply consider how to make achievement of radiative cooling better serving practice in the future.

## 1 Introduction

Cooling, as a part of thermal management, plays an important role in human activities, such as preservation of food and medicine for production and life and noise reduction for scientific studies. Air conditioners and electric fans are widely considered as representative of modern cooling technologies. According to the report in 2018, they account for nearly 20 % of the electricity consumed in buildings around the world [1]. This demand is set to keep growing. In 2020, cooling demand has already tripled globally since 1990 [2]. To meet cooling demand, over 2000 TW h (TWh) of electricity are ready to use every year [1]. Behind this figure, extremely large amounts of fossil energy are consumed, further aggravating global warming due to the release of heat and green-house gas [3]. It is inevitable to need more cooling, and so on in a vicious cycle [4]. If left unchecked, the predicted energy required for cooling will increase another twice by 2050 [1]. Facing this situation, the desire for renewable cooling is almost certain, for sustainable development [5]–[7].

Radiative cooling is a renewable cooling method proposed as a solution to mitigate global warming. The core concept is to reduce solar absorption and enhance radiative heat flow to outer space by specific spectral characteristics [8]. This is a passive process without external energy consumption, avoiding the results of local heat transfer but global internal energy increase, like air conditioners. In the recent decade, radiative cooling has greatly advanced with the development of photonics [9]. As the studies are going on, the commercialization of radiative cooling has been done without a stop, although it is in an early stage of development [10]. There is a blind spot in the existing literature. The reported research works are mainly limited to the cooling performance of materials and devices in the laboratory and give a broader assumption in the possible application scenarios that is often described by a simple sentence. But in fact, the barrier is not easily broken down by an amplification of samples with elaborate structure in the ideal conditions, when promoting radiative cooling from the laboratory to the practice. Besides technological challenges during scalable production, real effect, cost-effectiveness, and other problems could not be ignored. As a consequence, we proposed our own opinion about how to apply radiative cooling in the real world for everyone’s criticism.

In this perspective, we first review the history of radiative cooling briefly, clearly showing the dialectic relationship between theory and practice during the developing process. Then, based on the photonics concepts, we analyze the thermodynamic process of radiative cooling and talk about the possibility and way to break the limit of cooling performance in theory. The following issue is how to realize optimal spectral characteristics on objective entities. The topic of how to exactly evaluate cooling performance is proposed and discussed in the next section. The confusion of measured results in the literature indicates that it is important and necessary to make a whole series of standard evaluation protocols like lithium-ion batteries and solar cells. Finally, we point out the problems that may be faced and the possible way forward when radiative cooling goes commercialization, from laboratory back to practical applications. The content is not constrained to review the reported potential applications. Overall, all of discussion is done around the subject that radiative cooling serves practice.

## 2 Brief history of radiative cooling

**Figure 1:**
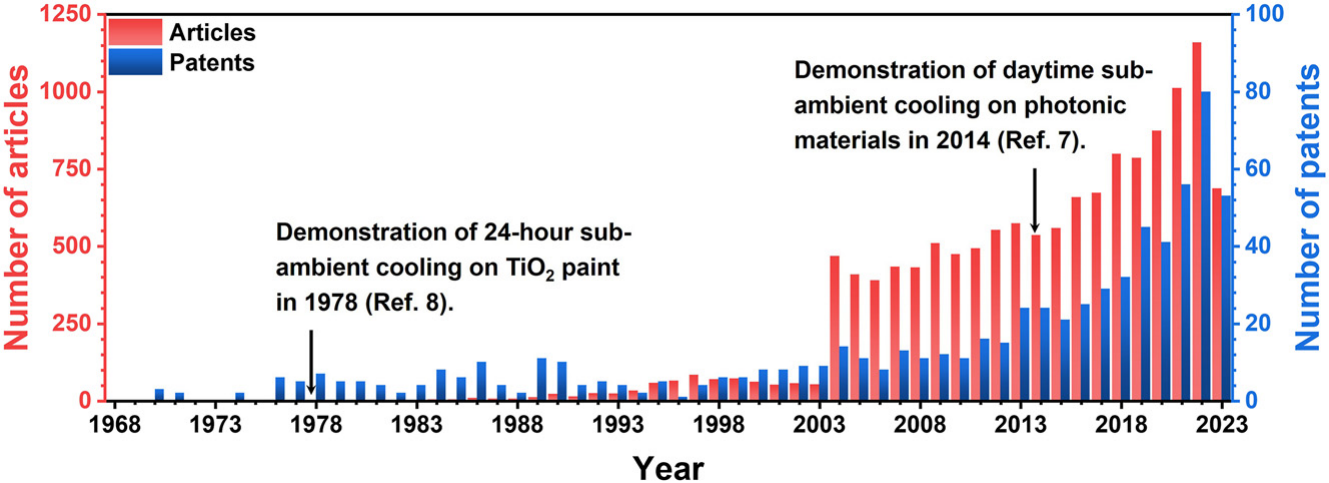
Articles and patents found containing “radiative cooling” in their topics. The search was done on the database “Web of Science” on September 20, 2023.

Exploration on radiative cooling has a very rich history. The applications of radiative cooling in practice could be traced back to thousands of years ago, long before systematic studies on it. The archaeological discoveries reveal that the ancient Iranians have constructed ice making basins and yakhchāl (namely ice pit) to produce and store ice even though it was a hot summer, since 400 BCE [11]. Reported by Eriksson and Granqvist, the first scientific studies on radiative cooling phenomenon were done by Arago in 1828 [12]. In 1962, Head proposed the concept of selective radiation for refrigeration, which starts the systematic studies of radiative cooling in theory and experiment [13]. This period of studies focuses on the designs to enhance nocturnal radiative cooling and its various applications. As mentioned by Bijarniya, the concept of passive daytime radiative cooling was first proposed by Trombe in 1967 [14], yet daytime subambient cooling was not realized in experiment until Fan’s work was reported by Nature in 2014 [15]. Another view is that daytime subambient cooling was first demonstrated by Harrison on titanium dioxide (TiO_2_) white paint [16], [17]. No doubt, booming studies on radiative cooling in the recent decade began at Fan’s work based on nanophotonic engineering and metamaterials technology (Figure 1). After that, various daytime subambient radiative coolers with different photonic designs, such as multilayer structure [15], randomly distributed particle structure [18], [19], and porous structure [20]–[23], have sprung up. While the cooling performance is improved continually, the applications of radiative cooling is also expanded greatly, including but not being limited to energy-efficient buildings [24], [25], enhanced solar cells [26], personal thermal management [27], electricity generation [28], [29] and water harvesting [30], [31]. Among them, dynamic thermal management [32], [33], especially dual-mode thermal management (including heating and cooling modes) [34]–[36], is born from static radiative cooling recently, in order to be against fluctuation in the aspects of space, time, day and season, temperature etc., in real practice.

The cognitive process of humans for radiative cooling is experienced from the perceptual into the rational, and from the practice to the theory. And then, the establishment of new theories drives the development of radiative cooling in theory and practice. As mentioned above, the ancient Iranians could produce and store ice by using naturally occurring radiative cooling. But until the finding of radioactive phenomenon, the researchers are just beginning to realize that radiative cooling is a radiative phenomenon and try to make in-depth analysis based on radiative theory. The discovery of atmospheric window motivates the researchers to consciously design cooler with selective spectral characteristics to enhance cooling performance [12], [37]. And the latest breakthrough is generated from the developments of photonic engineering [15]. Benefitted from getting rid of the designing dependence on natural objects, daytime subambient cooling comes true, beyond nocturnal cooling. Nowadays, the studies on radiative cooling are further divided according to different applications. The next breakthrough is likely to be born from them.

## 3 Fundamental limit of radiative cooling

Keeping improving cooling ability is always a challenging and urgent task to fully utilize radiative cooling in a wide application field. An important open question reasonably be thought then is: what is the fundamental limit of cooling ability? Two parameters are usually used to evaluate cooling ability. One is temperature reduction, and the other is net cooling power. They are linked by a thermal balance relationship, based on that the radiative cooler is exposed to a clear daylight sky [8]. The net cooling power of the cooler (*P*_net_) is given by

(1) $$\begin{array}{cc} P_{\text{net}}\left(T_{\text{cooler}},T_{\text{amb}}\right) & =P_{\text{cooler}}\left(T_{\text{cooler}}\right)-P_{\text{sun}}-P_{\text{atm}}\left(T_{\text{amb}}\right)-P_{\text{parasitic}}\left(T_{\text{amb}},T_{\text{cooler}}\right) \end{array}$$

There are four simplified objects included in this dynamic thermal balance, which are radiative cooler, the sun, outer space, and the ambient, respectively. Here, *P*_cooler_(*T*_cooler_) is spontaneous thermal radiation of the cooler, *P*_sun_ is absorbed solar radiation of the cooler, *P*_atm_(*T*_amb_) is absorbed thermal radiation of the cooler from the atmosphere, and *P*_parasitic_(*T*_amb_, *T*_cooler_) is the parasitic heat, namely heat exchange between the cooler and the ambient by heat conduction and heat convection.

An assumption is made that a radiative cooler is located in an open space and faces the sky with its normal direction pointing to the zenith direction. Thus, its spontaneous thermal radiation (*P*_cooler_) is determined by the temperature (*T*_cooler_) and the emissivity (*ε*_cooler_(*λ*, *θ*)) simultaneously.

(2) $$P_{\text{cooler}}\left(T_{\text{cooler}}\right)=A_{\text{cooler}}\int \text{d}\Omega \text{c}\text{o}\text{s}\theta \int_{0}^{\infty }d\lambda I_{\text{BB}}\left(T_{\text{cooler}},\lambda \right)\varepsilon_{\text{cooler}}\left(\lambda ,\theta \right)$$

*A*_cooler_ is the area of the cooler, Ω is a solid angle, and *θ* is the angle between the direction of the solid angle and the normal direction of the cooler surface. In doing so, $\int d\Omega =2\pi \int_{0}^{\pi /2}d\theta \sin\theta$ is the angular integral over a hemisphere. $I_{\text{BB}}\left(T_{\text{cooler}},\lambda \right)=\frac{2hc^{2}}{\lambda^{5}}\frac{1}{e^{\frac{hc}{\lambda k_{\text{B}}T_{\text{cooler}}}}-1}$ is the radiative intensity from a blackbody at temperature *T*_cooler_, where *h* is the Planck’s constant, *c* is the velocity of light, *k*_B_ is the Boltzmann constant, and *λ* is the wavelength.

The solar absorption (*P*_sun_) is a main heat source causing the temperature rise of the cooler, which is given by

(3) $$P_{\text{sun}}=A_{\text{cooler}}\overset{\infty }{\underset{0}{\int }}d\lambda \varepsilon_{\text{cooler}}\left(\lambda ,\theta_{\text{sun}}\right)I_{\text{sun}}\left(\lambda \right)$$

*I*_sun_(*λ*) is the intensity of the incident solar radiation. *θ*_sun_ is the angle between the direction of the incident solar radiation and the normal direction of the cooler’s surface.

Radiative heat absorption of the cooler from the ambient (*P*_atm_(*T*_amb_)) is a non-negligible component for the temperature rise.

(4) $$P_{\text{atm}}\left(T_{\text{amb}}\right)=A_{\text{cooler}}\int \text{d}\Omega \text{c}\text{o}\text{s}\theta \int_{0}^{\infty }d\lambda I_{\text{BB}}\left(T_{\text{amb}},\lambda \right)\varepsilon_{\text{atm}}\left(\lambda ,\theta \right)\varepsilon_{\text{cooler}}\left(\lambda ,\theta \right)$$

The angle-resolved emissivity of the atmosphere is *ε*_atm_(*λ*, *θ*) = 1 − *t*_atm_(*λ*)^1/cos*θ*^, with *t*_atm_(*λ*) being the transmissivity of the atmosphere in the zenith direction. *T*_amb_ is the temperature of the air around the cooler.

Nonradiative heat exchange (*P*_parasitic_(*T*_amb_, *T*_cooler_)) here, including heat conduction and heat convection, is a simplified model, considering all external objects that the cooler is in contact with as well as adjacent air.

(5) $$P_{\text{parasitic}}\left(T_{\text{amb}},T_{\text{cooler}}\right)=A_{\text{cooler}}h_{\text{c}}\left(T_{\text{amb}}-T_{\text{cooler}}\right)$$

It is linearly related to a combined nonradiative heat exchange coefficient (*h*_c_), which describes the collective effect of conductive and convective heat exchange.

The above thermal balance relationship reveals that the emissivity/absorptivity plays the central role in the thermodynamic process, and further essence of radiative cooling is photothermal conversion. In terms of spectral characteristics, there are three main aspects to improve cooling performance, especially subambient cooling, which are enhancing infrared emission, suppressing solar absorption, and reducing atmospheric absorption.

### 3.1 Nocturnal cooling and daytime cooling

The spectrum of solar radiation is similar to that of blackbody radiating at ∼5800 K and its power is considerable. The “solar constant” is roughly 1360.8 ± 0.5 W/m^2^ measured on the top of the atmosphere in 2008 [40], leaving maximum normal surface irradiance at approximately 1000 W/m^2^ at sea level on a clear day [38]. It mainly concentrates in the wavelength range from ultraviolet to near-infrared (from 0.15 μm to 2.5 μm). Nocturnal cooling could be seen as a special daytime cooling, where solar radiation is zero. So, for only nocturnal cooling, there is no specific demands on the solar absorptivity of the cooler. To achieve daytime cooling, it is essential for cooler to have a solar absorptivity less than 10 %, because the maximum infrared radiation power at room temperature is ∼100 W/m^2^, approximately equal to internal energy increase conversed from 10 % solar absorption. Taken together, the cooler needs to have a minimum solar absorptivity to suppress energy input from solar absorption.

### 3.2 Infrared selective cooler versus infrared broadband cooler

**Figure 2:**
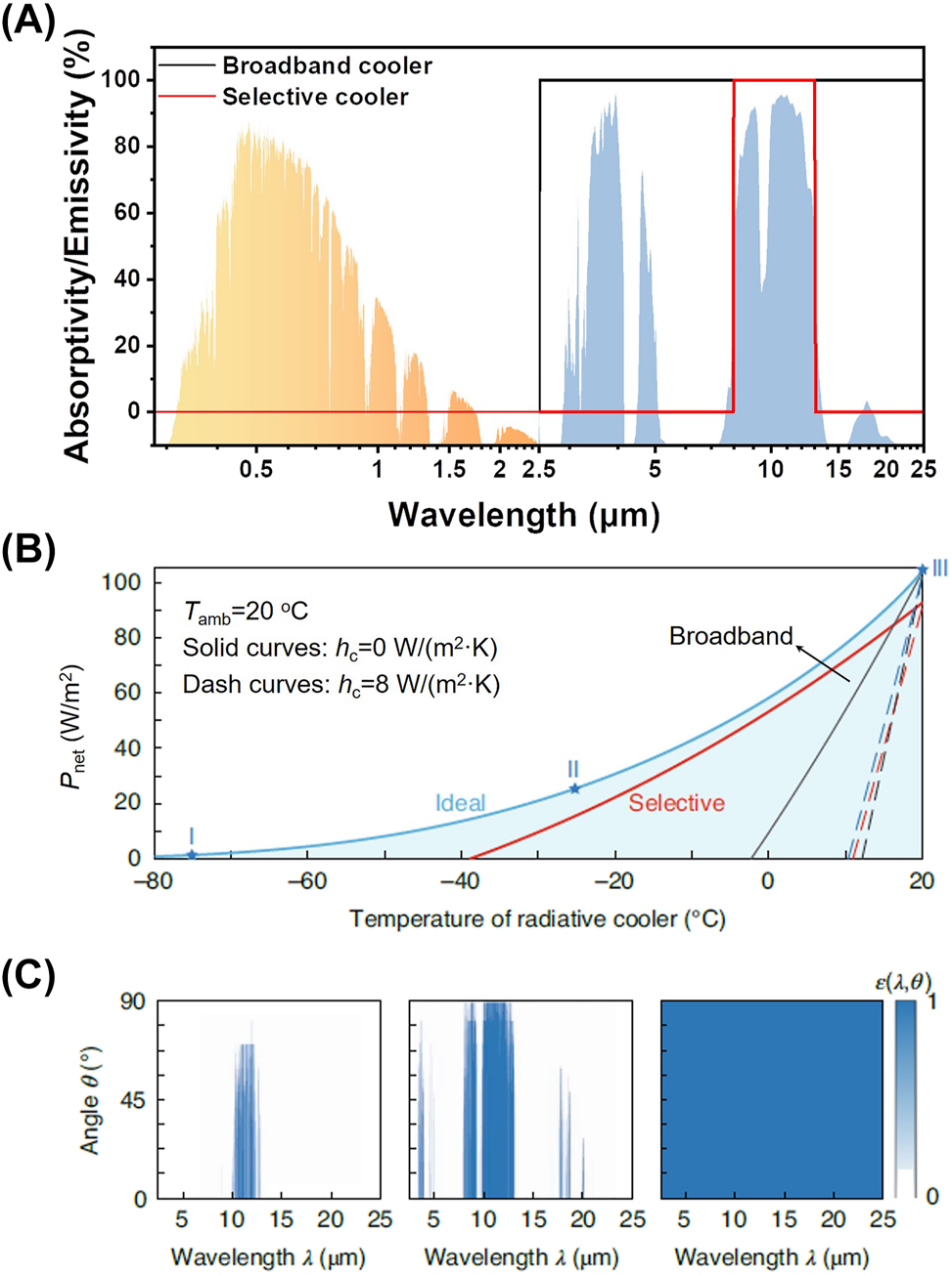
Theoretical analysis of cooling performance for (daytime) radiative cooling based on spectral characteristics. (A) Solar spectrum (normalized ASTM G173 Global solar spectrum [38], orange shaded area) and the atmosphere transmission spectrum in the infrared wavelength range (US standard 1976 [39], blue shaded area). The atmospheric transparent window at 8–13 μm has a large spectral overlap with the blackbody radiative characteristics at room temperature near 300 K. Infrared selective cooler (an emissivity of 100 % at 8–13 μm and zero emissivity at other wavelengths, red solid curve) and infrared broadband cooler (an emissivity of 100 % at >2.5 μm and zero emissivity at other wavelengths, black solid curve) have higher temperature reduction and stronger net cooling power, respectively. (B) Net cooling power (*P*_net_) as a function of radiative cooler temperature (*T*_cooler_) under various infrared emissivity profiles (*ε*_cooler_(*λ*, *θ*)), including the 8–13 μm infrared selective cooler (red), infrared broadband cooler (black) and optimal spectral-angular-selective cooler (blue). The light-blue shaded region is the range where optimal spectral-angular-selective cooler could operate. Part of labels have been modified based on description in this paper, referring to the literature [8]. (C) The optimal emissivity profiles of the radiative cooler aiming at different operating goals (blue stars on the blue curve in (B)) [8]: to achieve a lowest equilibrium temperature (left); to maintain the radiative cooler at a certain temperature that is below the ambient temperature while maximizing the net cooling power (middle); and to maintain the radiative cooler at or above the ambient temperature while maximizing the net cooling power (right). Panels adapted with permission from Springer Nature Limited.

At room temperature, the thermal radiation of objects is mainly infrared radiation, overlapping to atmospheric radiation. The infrared spectrum of the cooler has to be delicately designed, considering to enhance intrinsic infrared radiation and reduce atmospheric absorption together. Depending on spectral characteristics, infrared emission could be categorized as either selective or broadband. A spectral selective cooler has an emissivity of unity in the wavelength range of 8–13 μm, aiming at the maximum transparent window of atmosphere at room temperature. On the contrary, a broadband cooler (also called blackbody cooler) has 100 % of emissivity in the wavelength range >2.5 μm. Of course, two types of coolers both are assumed to have no solar absorption, namely 0 % of absorptivity in the wavelength range <2.5 μm (Figure 2A). Figure 2 shows the plots of the net cooling power as a function of the temperature of the coolers with different infrared spectral characteristics. The lowest temperature could be reached when the net cooling power is zero, so strongly influenced by nonradiative heat exchange. The net cooling power is the intersection corresponding to the temperature of the cooler equal to the ambient. In this situation, the cooler does not have nonradiative heat exchange with the ambient. This conclusion guides the measurement of cooling performance in experiment, which will be discussed in Section 5.2 in detail. From the cooling power–temperature plot, there is a clear difference that infrared selective cooler could reach a lower temperature, while the broadband cooler has a stronger net cooling power, under the same conditions. The selection of selective and broadband cooler is a game between intrinsic infrared emission and atmospheric absorption. Infrared radiation is a dynamic process, which power is positively associated with the temperature of the objects Equation (2). For subambient cooling, the infrared radiation of the cooler is less than the atmospheric absorption, in the range outside 8–13 μm. Thus, infrared selective cooler is easy to reach a lower temperature but has less cooling power. With the increasing temperature, infrared radiation gradually exceeds atmospheric absorption in the range outside 8–13 μm, and this gap widens rapidly. When cooling a heat source (often called above-ambient cooling), such as fluidic hot heat exchange media, broadband cooler has a better cooling effect. In general, to reach a low equilibrium, temperature requires an infrared selective cooler, whereas to have a large cooling, power requires an infrared broadband cooler.

### 3.3 Trade-off of ideal infrared spectrum

Further, the selective infrared characteristics could be optimized in consideration of the wavelength and angular dependency of a given atmosphere transmission spectrum [8], [41], [42]. The cooler with a narrow-band angle-selective profile (left in Figure 2C) will reach the lowest temperature possible in theory (Figure 2B). When the purpose is to maximize net cooling power at the same time that the cooler is at a certain temperature below the ambient temperature, the spectral characteristics of the cooler prefer to have a more complex profile (middle in Figure 2C). The cooler with a high emissivity in infrared broadband (right in Figure 2C) has a better cooling effect for high-temperature objects, just like the above description. Depending on the complexity of atmosphere, the optimization of infrared emission has to be elaborately tuned in every angle and wavelength. Although optimized cooler could be constructed in a small size in the laboratory, it almost has no chance to large-scale production and wide application in practice. In addition, the short-term weather and long-term climate are dynamic, which makes a great impact on temperature reduction of the designed cooler based on a given atmosphere transmission spectrum. Therefore, the cooler with selective spectral characteristics has more advantages than that with ideal spectrum in the real application. Even so, the analysis of ideal spectrum is also instructive to design radiative cooler. For example, selective cooler is a compromise of narrow-band angle-selective profile (left in Figure 2C) to the practical scenario.

### 3.4 Reconstruction of spatial structure

Besides optimizing the intrinsic spectral characteristics of the cooler, the geometric parameter (*A*_cooler_) could be tried to build something around to improve the radiative cooling performance. The above analysis of thermal balance relationship is done based on an assumption that could not be ignored, where a radiative cooler is located in an open space and faces the sky with its normal direction pointing to the zenith direction. In doing so, the effective area for radiative cooling is equal to the area of the cooler exposed to the sky. If activating the rest of unexposed area, the cooling performance per unit area, no matter temperature reduction and cooling power, can significantly improve, because of the fixed calculated area. Zhou et al. have demonstrated this idea in theory and experiment [43]. Activating unexposed area is an indirect way to enhance infrared emission of the cooler.

### 3.5 Influence of nonradiative heat loss

Parasitic heat loss from nonradiative heat exchange has no impact on the intrinsic radiative cooling performance of the cooler, but it will directly influence the measurement results in experiment. This loss, of course, weakens the cooling ability of radiative cooler in practice. The effect of nonradiative heat exchange is discussed around the thermal management evaluation protocols in Section 5.2 in detail.

Limited by our knowledge, it is inevitable to omit some advanced research results by mistake. However, pursuing the goal of closing to and even breaking the limit of radiative cooling is never ended. Every breaking is born of the advances in photonic science and technology. Sometimes, a few breakthrough cooling performances could be realized under strict conditions. But only in terms of applications, we have to pay more attention to the practical of designed radiative cooler in balance with spectral characteristics.

## 4 Selection of raw materials and design of geometrical structure

For applied studies, the analysis and optimization of spectral characteristics in theory are destined how to realize them on objective entities. Selection of raw materials and design of geometrical structure are two main respects for realization of this purpose. There are many published reviews continuing to summarize the latest advancement of radiative materials in detail from many different perspectives. In this section, we focus on the summary of evolution law and essential milestones in it, rather than the concrete content of any work. Selection of raw materials and design of geometrical structure complement each other in making process of radiative cooling. We could not and should not look at them in isolation simply.

### 4.1 Selection of raw materials

Polymers are investigated as promising candidates for radiative cooling materials in the early stage. Diverse feature structures and functional groups, covering broad infrared wavelength range, allow for immense possibilities of spectral design [44]. Among them, polyethylene (PE) and nylon are often studied for infrared transparent materials, due to their simple structure [45]. Especially PE, it has been widely used as solar- and infrared-transparent shields in measurement of cooling performance for radiative cooling. Most polymers are used as infrared absorbers. Modification treatment could further enrich polymer’s intrinsic infrared radiation, by chemical introduction of other functional groups.

Metals often have low absorptivity, particularly in the infrared wavelength range. Metal film needs just about one hundred nanometers of thickness to reflect more than 90 % of infrared radiation. Thus, metal layer is often used as back of radiative coolers [18]. The most common metals are silver (Ag) and aluminum (Al), because of lower solar absorption and cost. Ag has a better solar reflection effect than Al but is more expensive too. They could be selected according to the comprehensive consideration in different scenarios.

Inorganic nonmetal materials, a broad category of alternative bulk materials, have been investigated extensively in the pioneering works. Titanium dioxide (TiO_2_) [19], [46], aluminum oxide (Al_2_O_3_) [22], [23], [47], [48], silicon dioxide (SiO_2_) [18], barium sulfate (BaSO_4_) [49], boron nitride (BN) [50], and so on are frequent here. Recently, perovskite crystals are also used to construct radiative cooling materials [51]. One common usage is to fill inorganic nonmetal materials into polymer matrix. Large difference of refractive index between fillers and matrix enhances the scattering effect of composites for solar radiation and makes them exhibit high solar diffusive reflection. Inorganic nonmetal materials could also be used as matrix directly. Materials with porous structure have similar high solar reflection, due to the difference of refractive index between inorganic nonmetal materials and air. Rutile TiO_2_ has the highest refractive index among them but absorbs a lot of ultraviolet radiation. BaSO_4_ is the most typical coating used in integrated sphere for ultraviolet-visible-near-infrared (UV-VIS-NIR) spectrometer. BN processes higher thermal conductivity. Every inorganic nonmetal material has its special properties, which is chosen according to the real applications.

The scientists have also considered fluidic radiative cooling materials, including gas and liquid. The representative is ammonia (NH_3_) [52]. After that, ethylene (C_2_H_4_), acetaldehyde (C_2_H_4_O), and others are investigated successively [53]. Today, utilization of gaseous-state radiative cooling materials is embodied in porous radiative cooling materials [20], and the liquid is used in dynamic radiative cooling [54].

Overall, selection of raw materials is mainly dependent on their intrinsic electromagnetic properties. Good selection will greatly relieve stress for following design of geometrical structure. Besides electromagnetic properties, other requirements from practical application have to be considered simultaneously. The core thesis in this section is the influence of raw materials on spectral characteristics, so there is not much discussion on other requirements.

### 4.2 Design of geometrical structure

Nowadays, there is no known single raw material meeting the entire demands on spectral characteristics for radiative cooling. So, design of geometrical structure becomes very important to enrich and combine spectral characteristics of different raw materials and then construct functional materials with optimized selective spectrum.

**Figure 3:**
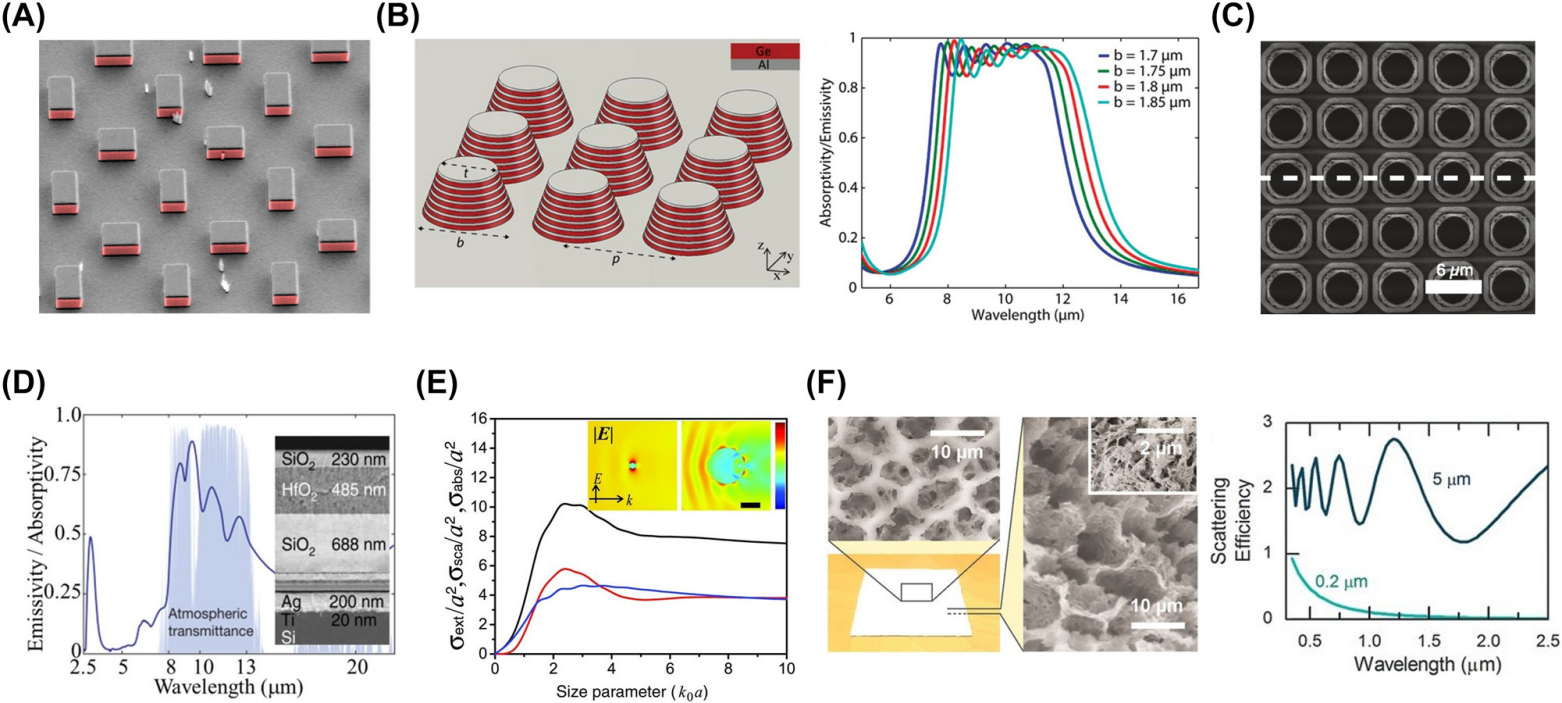
Design of geometrical structure in radiative cooling materials. (A) Wide-angle broadband infrared absorption based on magnetic dipole resonance of dielectric resonator [55]. (B) Array of symmetrically shaped conical multilayered pillars for selective infrared emission (left) and corresponding calculated spectra for different bottom diameters of the pillars (right) [56]. (C) Two-dimensional SiO_2_ photonic crystal with a periodic square-lattice structure for enhancement of infrared emission [26]. Panels A–C show the application of periodic configuration on radiative cooling. (D) Aperiodic multilayers composed of alternative SiO_2_ and HfO_2_ layers with different thicknesses for selective infrared emission [15]. (E) Strong phonon–polariton resonances of SiO_2_ sphere with a diameter of ∼4 μm to enhance infrared radiation at 9.7 μm in poly(4-methyl-1-pentene) (PMP) doped by SiO_2_ [18]. (F) Scattering effect of voids in porous structure (left) for enhancement of global solar reflection (right) [20]. Panels D–F show the application of breaking geometric symmetries on radiative cooling. Panels adapted with permission from Wiley Online Library, National Academy of Science, Springer Nature Limited, and AAAS.

Periodic configuration has been widely studied on constructing radiative cooling materials. Homogeneous bulk is the simplest form with periodic configuration. Multilayered, arrayed, or combined structures are focused for a certain period of time (Figure 3) [26], [55]–[57]. This strict translational symmetry in periodic configuration is difficult to be realized in engineering, but it gives extra freedom in the design with breaking geometric symmetries. The common breaking geometric symmetries on radiative cooling materials are aperiodicity and randomness [58]. For example, aperiodic multilayers composed of alternative SiO_2_ and hafnium (IV) oxide (HfO_2_) layers with different thicknesses limit the infrared radiation in the atmospheric window of 8–13 μm [15]. The SiO_2_ sphere with specific dimensions exhibits strong high-order resonances at the extinction peak, which resonances fill in gaps of infrared radiation in the atmospheric window of 8–13 μm [18]. The mechanism of porous structure is to enhance the scattering for solar radiation, due to numerous and randomly distributed air voids with different dimensions. The final result is high solar reflection [20]. During this process, there are at least two raw materials (including air) used to construct radiative cooling materials. The structure with aperiodicity and randomness, by contrast, is easier to achieve in manufacture engineering and thus attracted more attention recently.

## 5 Practices on criteria for cooling performance

For the last decade, the number of studies on daytime subambient radiative cooling has increased rapidly, but an issue could not be ignored is that the results from nonunified estimation methods make it difficult to do a compelling comparison on cooling performance for objectively showing the studying advancement of radiative cooling. Further, this issue hinders the application of radiative cooling in practice. Hence, it is important, imperative, and urgent to make a series of standard evaluation protocols against specific performance metrics and rigorous experimental steps.

### 5.1 Spectral characteristics and prediction of cooling performance

The detailed analysis of thermal balance relationship indicates that we could predict temperature reduction and cooling power of any radiative cooler based on its spectral characteristics and given ambient conditions (including solar spectrum, atmospheric spectrum, and nonradiative heat exchange coefficient). Due to the uniform physical model and rigorous mathematical deduction, the prediction is a fair criterion for evaluation of cooling performance between radiative coolers. The difference between them is their own spectral characteristics and the following challenge is how to accurately characterize the electromagnetic spectrum by a fair measuring standard.

The spectral range needs to cover the whole solar radiation and infrared emission of blackbody, such as wavelength from 0.25 μm to 2.5 μm for solar radiation and from 2.5 μm to 20 μm for thermal emission. It is crucial, because the deviation of predicted cooling performance will be amplified with the lack of information in energy-concentrated range of solar and infrared radiation. The data could already be characterized by mature and commercial spectrometers. There are three details worth noting. First, the calibration is a key step before measuring. The selection of standard reference directly determines the credibility of measured spectra. Second, the global spectrum is required, namely measured by spectrometers with integrated sphere, especially for high-scattering materials. In some special application scenario, polarization or angle-dependent spectrum may also be necessary, for example, directional thermal radiation. Finally, it is still a challenge to obtain the whole spectrum across the wavelength range covering solar to infrared radiation in a continuous scanning measurement, due to the technical limitations of existing commercial spectrometers. The alternative way is to combine several spectra across different wavelength range measured by different spectrometers. In doing so, line breaks are often observed in the connects of adjacent spectra. This technical issue is often addressed by calibration likewise.

The analysis of spectral characteristics is a complex process. We could simplify the whole spectrum into several representative indices, such as weighted-average solar reflectivity and infrared emissivity in atmospheric window (8–13 μm) [59]. But the simplifying treatment is a double-edged sword, leading to shortcut of estimation and lack of information. After all, ignored information, sometimes, will be the determining factor, for example, infrared broadband cooler is better than infrared selective cooler for cooling high-temperature objects. However, the comparison of spectral characteristics is still a convenient and universal way to estimate cooling performance under a premise of integrity and credibility of measuring spectrum.

### 5.2 Measurement of cooling performance

Admittedly, spectral characteristics of radiative cooler at a specific wavelength range are key physical parameters describing dynamic photonic-thermal conversion in radiative cooling. While the potential cooling performance, including temperature reduction and net cooling power, could be estimated from spectral characteristics, experimental thermal measurement is an indispensable part of cooling evaluation in practice. After decade of vigorous development, the measured spectral characteristics of radiative cooling materials with well-designed structure are becoming more and more like the ideal selective spectrum in theory as well. Making a comparison for the reported works, a series of contradictions that we have to acknowledge emerge, where (i) the temperature reduction of materials with near-ideal spectral characteristics is lower than that with the bad, (ii) the materials have a higher temperature reduction but a poor cooling power, and (iii) the materials exhibit a counterintuitive cooling performance or could not realize a daytime subambient cooling result. Nonuniform measuring method and reference result in the above contradictions. Hence, rigorous and standard thermal management evaluation protocols are necessary.

At the beginning of Section 3, we mention the parameters to evaluate cooling ability: temperature reduction and net cooling power (Figure 2B). The specific definition of temperature reduction is the reducing temperature of the cooler compared with the ambient (Δ*T* = *T*_amb_ − *T*_cooler_). The lowest temperature for a given cooler could be achieved when the net cooling power is zero. The measured result is sensitive to the comprehensive heat exchange coefficient (*h*_c_). In other words, the measurable temperature reduction is influenced by the thermal insulation effect of the measurement system from the environment. Therefore, it is a one-sided view to evaluate cooling performance by temperature reduction alone. Another parameter is net cooling power, which is the intersection corresponding to the temperature of the cooler equal to the ambient in the plot. Guided by this conclusion, the cooling power of the cooler is measured in experiment when its temperature is the same as the ambient.

**Figure 4:**
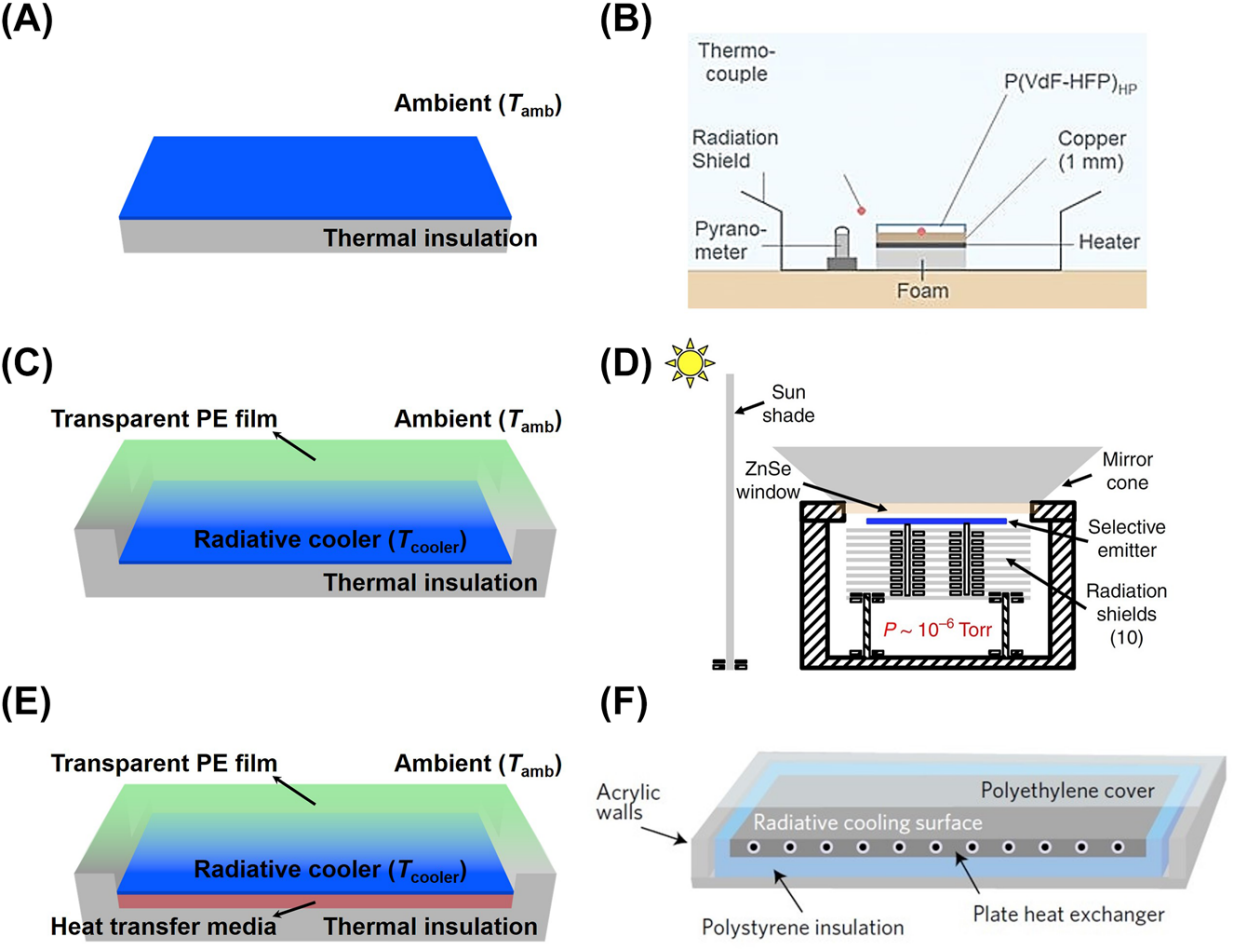
Three different thermal measurement methods for radiative coolers. (A) Radiative cooler (blue) is placed on a thermal-insulated substrate (gray) and directly exposed to the ambient. (B) The application of measurement method (A) in the literature [20]. (C) Radiative cooler (blue) is placed in a thermal-insulated enclosure (gray) with a transparent PE film (green). (D) The application of measurement method (C) under the high vacuum in the literature [61]. (E) Radiative cooler (blue) is placed on an object (such as fluidic heat transfer media, orange red) to demonstrate the effective utilization of generated cooling capacity. (F) The application of measurement method (E) in the literature, where heat transfer media is water [64]. Schematic illustrations (A, C, and E) have been redrawn referring to the literature [60]. Panels adapted with permission from AIP Publishing, AAAS, and Springer Nature Limited.

Three types of measurement methods with the schematic are shown in Figure 4 [60]. The first one is that a radiative cooler is directly exposed to the ambient, which is usually for those applications to cool the underlying structure, such as a cool roof (Figure 4A and B). When a thermal insulation is instead of the underlying structure, the measured results imply the maximum cooling performance of the radiative cooler in a real scenario [20]. On this basis, the radiative cooler is sealed in a well-insulated enclosure with a transparent polyethylene (PE) film (Figure 4C and D). This transparent convection shield suppresses nonradiative heat transfer (namely heat conduction and convection loss) between the cooler and the ambient well, without influence on the transmission of solar radiation and infrared radiation. A large subambient temperature reduction could be achieved by using this design, which is usually employed to demonstrate the effectiveness of a wavelength-selective cooler. More dramatically, the thermal-insulated enclosure is replaced by a dedicated vacuum. Under the high vacuum, Chen et al. demonstrated an astonishing temperature reduction of ∼42 K from the ambient [61]. The measured results are very close to the theoretical prediction calculated when nonradiative heat exchange is zero. The above two measurement designs focus on demonstrating a temperature difference of the radiative cooler with the ambient, but with a small thermal mass. In practice, one might be more interested in cooling an object with a certain thermal mass, such as thermal transfer media. This measurement design shown in Figure 4E and F demonstrates the effective utilization of generated cooling capacity, instead of the temperature difference between the cooler and the ambient.

Besides the selection of thermal measurement methods, the measuring conditions also influence the experimental results. Compared with the dynamic nature in outdoor environment, the features of indoor environment are controllable more easily. In early practice, a tank with high infrared emissivity is filled with liquid nitrogen to provide a stable cold source at 77 K in indoor experiment [43], [62]. This case simulates a nighttime scenario with a clear sky, which could show the gap of cooling performance caused by different infrared emissivity. Recently, the researchers designed a complex setup integrated with simulated solar radiation and cold dome together [63]. What is more, a wavelength-selective filter mimics the selective screening effect like atmospheric transparent window. The impact of solar radiative intensity, ambient temperature, and state of the sky could be investigated in detail. This design vividly reconstructs the environmental conditions outdoors in an indoor setup, although there are still a few differences between them, such as more concentrated spectrum of solar radiation, lower temperature of outer space, more complex selective transmission of atmospheric window, and lack of atmospheric radiation. However, it is an important step in the development of a standardized test to bring accuracy, reproducibility, and comparability to the radiative cooling materials.

So far, indoor simulated test could not fully reflect the cooling performance of radiative coolers in the open air alone. Various ambient conditions are the most prominent characteristics of field test outdoors, which cause great difficulty in comparison of cooling performance between different radiative coolers reported in the literature according to results of field test. For example, there is a strong absorption of water molecules for infrared radiation. It means that the higher humidity is, the lower transmissivity of atmosphere in the infrared window is [65]. The clouds in the sky not only shield the solar radiation but also block infrared radiation from the radiative cooler [60], [66]–[68]. In addition, the definition of the ambient temperature, the relative location of the cooler with the sky and the sunlight, the materials forming the measurement design, and other factors all influence the final measured results. As a result, simplistic comparison on temperature reduction and net cooling power has little meaning to evaluate cooling performance of different coolers reported in the literature. Aiming at these problems, three scientists, Lyu Zhou, Xiaobo Yin, and Qiaoqiang Gan, give advice in the recent comment. These measures include ensuring temperature measured in a weather station with a radiative shield as ambient temperature, introducing a fair reference to do synchronous test, and measuring cooling power by using a feedback-controlled system [59]. For the goal of application, the experiment must be done considering the real scenario. Comparing the radiative coolers with existing commercial products under a comparable scenario is feasible.

### 5.3 Potential of energy saving or carbon reduction

Prediction of energy-saving or carbon-reduction potential is a practice that is estimating cooling power in a specific application scenario based on spectral characteristics. It is often done by establishing a scenario-based model or using a mature simulating software. Comparing with prediction of cooling performance (Section 5.1), this estimation is more targeted. For example, EnergyPlus modeling could evaluate energy-saving potential in a conventional air-conditioning utility based on long-term local weather data, when integrating proposed radiative coolers into building envelopes [21]–[23], [34]. No doubt, this evaluation is rough due to utilization of average statistic data, but its trends and laws are significant for optimization of radiative coolers. In addition, one important value of radiative cooling technology is energy saving and carbon-reduction. Therefore, estimation of energy-saving or carbon-reduction potential has become a necessary indicator, especially when emphasizing that reported radiative coolers has a huge potential in a specific application field.

## 6 From laboratory back to practical applications

After a series of theoretical and experimental studies, the new achievement of radiative cooling, especially daytime subambient cooling, is moving out of the laboratory and into practical applications. Although many reported works seem just short of investment away from commercialization, there is actually a considerable barrier between the studies in the laboratory and the applications in the real world. These include common problems in the process of commercialization and special problems just aiming to radiative cooling must be solved step by step. In this section, we will make a discussion on the possible problem during the process of commercialization, rather than a review about different applications of radiative cooling have been reported in the literature. Only our opinion is just for reference.

### 6.1 Combination with other cooling technologies

In Section 3, we have discussed the fundamental limit of radiative cooling in detail. However, the radiative cooling power is limited to a theoretical value of ∼100 W/m^2^ at room temperature [8], [61], although a cooling power beyond the theoretical value has been measured in a specific structure [43]. Besides, the high-performance radiative cooling in practice is heavily dependent on dry weather/climate, clear sky, and open space. Taken together, radiative cooling is more suitable to compensate, rather than replace active cooling in an integrated cooling system for reducing energy consumption, or cooperate with other passive cooling behaviors to enhance cooling performance in total.

#### 6.1.1 Integration into an active cooling system

**Figure 5:**
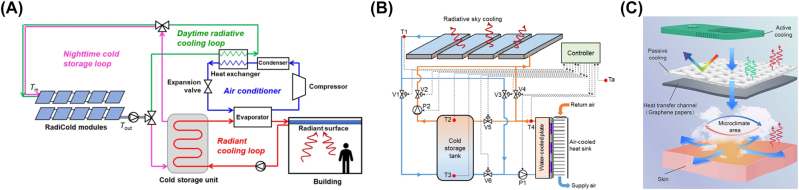
Representative integration of radiative cooling with active cooling system. (A) Initial cooling of fluidic heat-transfer media by radiative cooling. Cooling panels are integrated into air-cooled radiator by piping configuration [69]. (B) Hot side of thermoelectric cooling system cooled by generated cooling energy by radiative cooling [70]. (C) Multi-effect design with integration of thermoelectric cooling, radiative cooling, and high-performance heat conduction [71]. Panels adapted with permission from Springer Nature Limited and Elsevier Limited.

Reducing the energy demands of buildings on thermal management is an important application for radiative cooling. An easy way to do this is to cover the buildings with radiative cooling materials [25]. It is viable when a lonely building is located in the open air. But if a large number of buildings are crowded in the city, the effective area will decrease sharply. What is more, a possible negative influence is that the buildings absorb the infrared radiation from the surrounding heat sources due to its high infrared absorptivity, resulting in the aggravation of energy consumption for cooling. Referring to the design in the literature, the radiative cooling could be applied to initial cooling of fluidic heat-transfer media in active cooling system (Figure 5A) [64], [69]. The dominant active cooling technologies are well-established. The combination will significantly improve the efficiency in generated thermal energy use and reduce the demands on energy. Meanwhile, the cost and difficulty of technology transfer is less than designing a completely new cooling device. Another selection is to cool down the heat sink in an active cooling system (Figure 5B) [70]. It could enhance the cooling performance of active cooling technologies under the premise of same energy consumption, which indirectly reduces the energy demands. In some cases, active cooling and radiative cooling could be used to cool down a same object simultaneously (Figure 5C) [71]. This is a simple composition but also omitting the design on auxiliary equipment and operation mode. In general, it is a system-level approach to improve renewable energy generation and efficiency in the future.

#### 6.1.2 Cooperation with other passive cooling technologies

**Figure 6:**
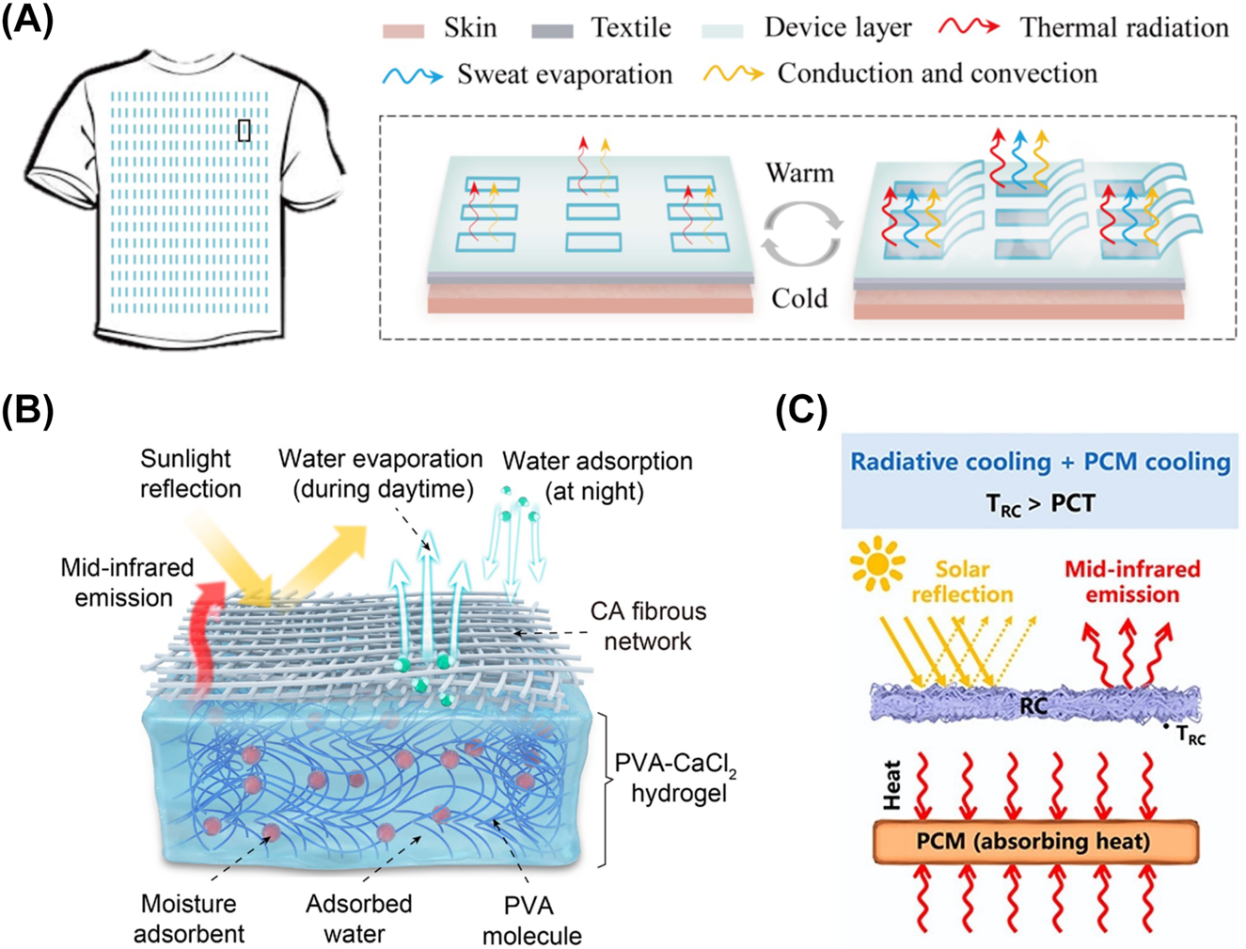
Representative combination of radiative cooling with other passive cooling technologies. (A) Multimodal adaptive personal thermal management, where regulating infrared emission, sweat evaporation, and thermal convection together [72]. (B) Tandem radiative/evaporative cooler for weather-insensitive passive cooling [73]. (C) Phase change materials enhanced radiative cooler for temperature-adaptive thermal management [74]. Panels adapted with permission from AAAS and American Chemical Society.

Passive cooling technologies rely on natural heat transfer phenomenon. Zero-energy consumption is the greatest merit of them, but it also comes with inevitable limits of natural conditions. In this context, researchers come up with the idea that the unity of different passive cooling technologies overcomes respective disadvantages (Figure 6) [72]–[75]. For example, weather conditions have a great impact on cooling performance of radiative cooling, which has been discussed repeatedly above. Evaporative cooling, another passive cooling technology, is limited by continuous water supply with complex systems. Li and his coworkers propose and demonstrate a tandem radiative/evaporative cooling system that exhibits excellent cooling performance under various conditions of weather/climate (Figure 6B) [73]. However, the studies on the cooperation of radiative cooling with other passive cooling are in an early stage of development.

### 6.2 Dynamic thermal management

In the real world, almost all the ambient scenarios come with the challenge that the objects are located in a quite dynamic and variable environment, including the fluctuation in the aspects of space, time, day and season, temperature, etc. Except for some high-power systems with large amounts of waste heat generation, a person or thing always works well in a temperature-comfortable environment. It means that fixed radiative cooling is not completely suitable for the dynamic ambient. From the temperature control perspective, unwanted cooling will increase the energy consumption for heating in the cold, and even may offset the energy saving of cooling in the hot. The proposal of dynamic thermal management, including modulable radiative cooling and dual-mode thermal management, basically begins here.

The essence of dynamic thermal management is the modulation of spectral characteristics. The expected one would be that the device could switch between heating and cooling modes, which maximums the utilization of both the inexhaustible radiative heat source (the sun, ∼5800 K) and cool source (outer space, ∼3 K) in the nature. More specially, for ideal heating mode, the materials should have high solar absorptivity and low emissivity in the wavelength range >2.5 μm, determined by the sunlight spectrum and blackbody radiation law [80]. For ideal cooling mode, especially daytime subambient radiative cooling, the materials are desired to efficiently reflect solar radiation (0.2–2.5 μm) and have strong selective mid-infrared emission in the specific wavelength range of transparent atmospheric window (8–13 μm) [61], discussed in Section 3 in detail. Modulable radiative cooling is approached as an incomplete dual-mode thermal management, where only a part of spectral characteristics is modulated.

**Figure 7:**
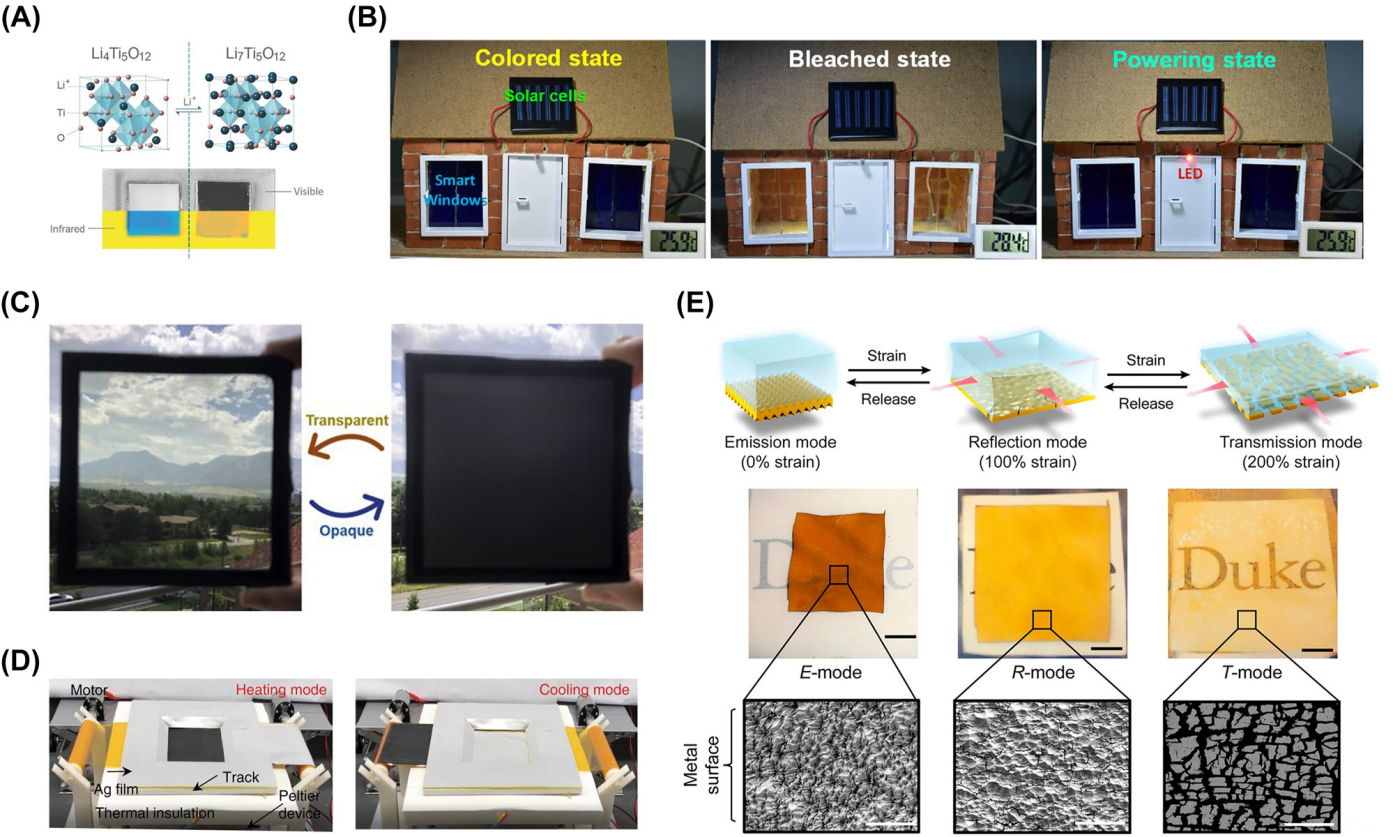
Representative dynamic thermal management with active modulation. (A) Crystal structure, optical and infrared images of Li_4+*x*_Ti_5_O_12_ nanoparticle layer switching between delithiated (DL) state (Li_4_Ti_5_O_12_) and lithiated (L) state (Li_7_Ti_5_O_12_) [76]. (B) Appearance evolution of a Prussian blue-based window between colored state (left) and bleached state (middle). A fully charged window could lighten a red light-emitting diode (LED) for tens of seconds [77]. (C) Reversible appearance evolution of dynamic window based on metal electrodeposition [78]. (D) Switch of functional thermal management materials driven by rolling rotation [34]. (E) Schematic illustrations (top), optical images (middle), and surface microstructures (bottom) of modulator reversibly switching from emission mode (left) to reflection mode (middle), and further to transmission mode (right) with different tensile strain [79]. Panels adapted with permission from Wiley Online Library, Elsevier Limited, Springer Nature Limited, and American Chemical Society.

According to whether energy consumption or not during the modulating process, dynamic thermal management could be divided into active modulation and passive modulation. Electrochromism [76], [77], electrodeposition [78], [81], [82], and mechanical deformation [34], [79], [83]–[86] are three representative strategies of active modulation (Figure 7). From the brief summaries of the above studies, the most outstanding characteristic of active modulation is that the trigger timing belongs to the administrator, not subject to the fluctuation of the ambient, but at the cost of external energy consumption and related low space utilization. External energy consumption, such as electric and mechanical energy, has to be lower than the energy saving, otherwise the meaning of radiative thermal management is insufficient. Meanwhile, some auxiliary equipment is used to apply external energy, resulting in the ratio reduction of thermal management materials in the whole design.

**Figure 8:**
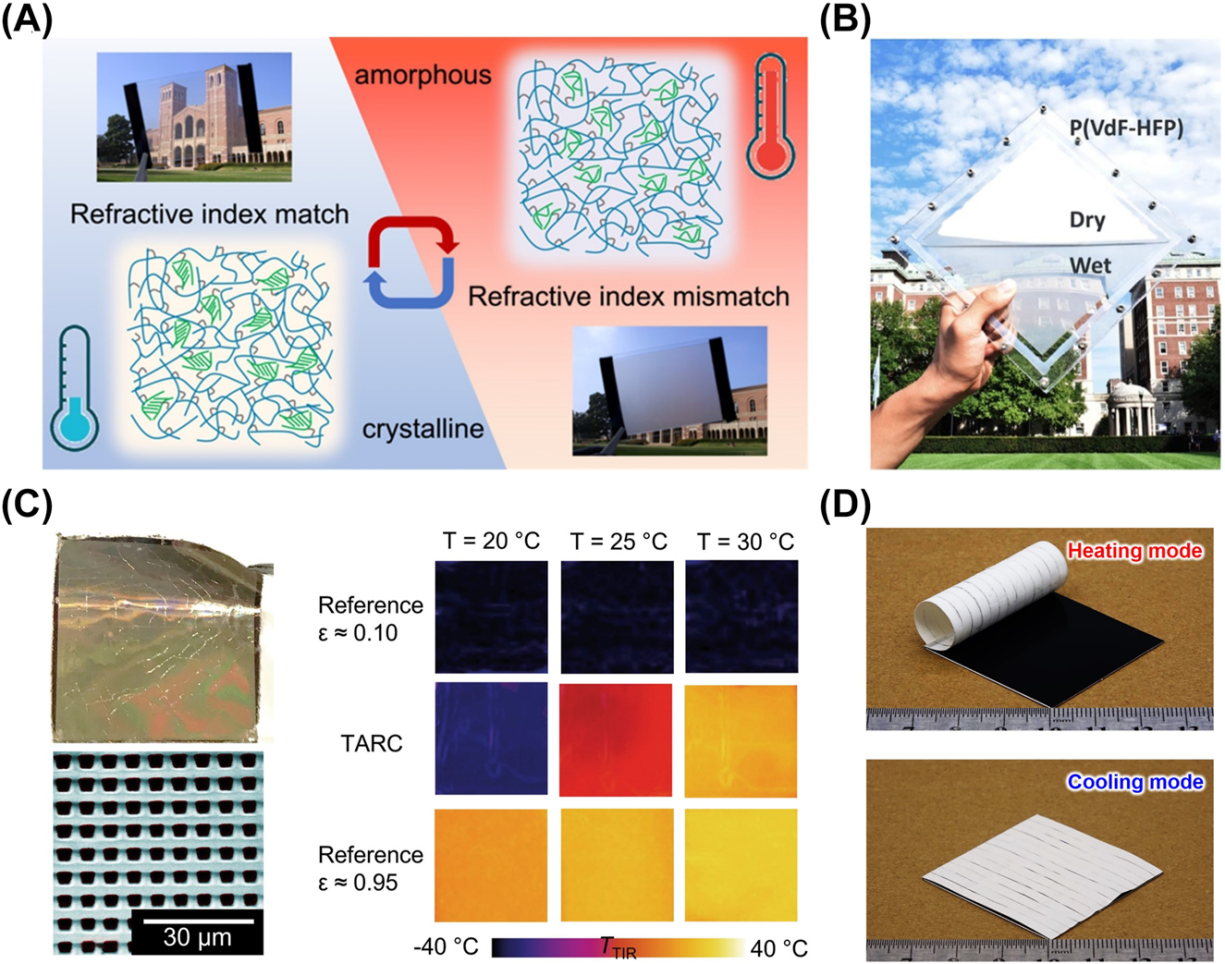
Representative dynamic thermal management with passive modulation. (A) Opacity of a shape memory film dependent on temperature-sensitive crystalline degree [92]. (B) Optical image of a liquid/porous film system at dry and wet states together [54]. (C) Optical appearance and microstructure of tungsten (W)-doped vanadium dioxide (VO_2_) coating (left). Compared with two conventional materials (references) with constantly low or high infrared emissivity, VO_2_ coating shows a temperature-sensitive infrared emissivity [32]. (D) Optical images of dual-mode thermal management device based on temperature-sensitive actuation at heating (top) and cooling (bottom) modes, respectively [35]. Panels adapted with permission from American Chemical Society, Elsevier Limited, AAAS and Springer Nature Limited.

On the contrary, passive modulation is the reaction to environmental change, such as temperature [35], [36], [87], humidity [72], [88], light [89], and other stimuli. Efforts toward a passive modulating system have been constructed based on thermochromic materials [90], phase-change materials [32], [33], [91], [92], liquid-porous polymer mixtures [54], shape-memory polymers [35], [36], and so on (Figure 8). Although now the response range could be designed by material modification, the trigger timing has been determined at the beginning of materials selection. It is remarkable that the concept of whole process zero-energy dual-mode thermal management is proposed in theory and demonstrated in experiment, at 2022 (Figure 8D) [35]. This design has two sets of completely high-selective spectral characteristics corresponding to heating and cooling modes, and the ability to temperature-dependent selection of thermal management modes. Combined with the previous reports [34], group of Ashraf Abedin gives their four criteria for dual-mode thermal management on this basis: (i) superior heating and cooling performance comparable to that of the majority of state-of-the-art solar heating and radiative cooling materials used alone; (ii) low material’s thermal resistance to maximize the generated heating/cooling power or temperature difference; (iii) flexible and light-weight properties to remain durable enough to perform after switching cycles; and (iv) minimized thermal contact resistance between the soft and flexible material and heated/cooled objects [93].

In general, active modulation is more a reflection of the temperature control intent from administrator to express, while passive modulation endows dynamic thermal management the real zero-energy consumption in the whole process including working and modulating. Up to now, these two types of modulation still have their own advantages and disadvantages. In addition, the studies on the modulation of spectral characteristics across solar and infrared radiation are still very insufficient, no matter active modulation or passive modulation. Even though, we consider dynamic thermal management to be an important development direction in the future.

### 6.3 Multifunction or “the skilled-art of slaughtering dragons – useless skill”

Apart from continuing to deepen understanding in theory and develop potential in practice of radiative cooling, it is also an important research field to endow radiative cooling materials more other functions. This study generates from two main reasons. One is expected that the extra performance is helpful to enhance cooling performance, enlarge application scope, or prolong service life. The other is that utilization rate of space is increased by adding extra functions on the platform of radiative cooler. For example, researchers attempt to make the radiative cooling materials hydrophobic [20], [94], [95]. Modified materials have a self-cleaning ability to antipollution, which could prevent decay on spectral characteristics due to dust attachment. It effectively prolongs the service life of corresponding materials. Another example is radiative cooling textile with directional water transportation [96]. The organic combination of evaporative cooling and radiative cooling improves the overall effect on cooling performance for better personal comfort. The above two examples show that each function should make the other better, rather than be a stack and even conflict with each other. The requirements come from the problems encountered in practice. Otherwise, there is no suitable application scenario requiring this useless design, although integrating these functions into an assembly is not easy. It is just like the Chinese story “the skilled-art of slaughtering dragons.” The hero masters the superior skill of slaughtering dragons after undergoing innumerable trials and hardships, but where is the dragon on the earth? The history shows a basic developing thread of radiative cooling that arises from practice, and in turn serves practice. We firmly believe that the advance of radiative cooling in the future should not and could not be away from the practice.

### 6.4 Potential and extension applications

At room temperature, daytime subambient radiative cooler could provide a net cooling power of around 100 W/m^2^ [8], [61]. The power is not strong enough in a few large-scale energy-intensive cooling scenarios, but generated cold is skillfully exploited for some potential and extension applications.

#### 6.4.1 Electricity generation

**Figure 9:**
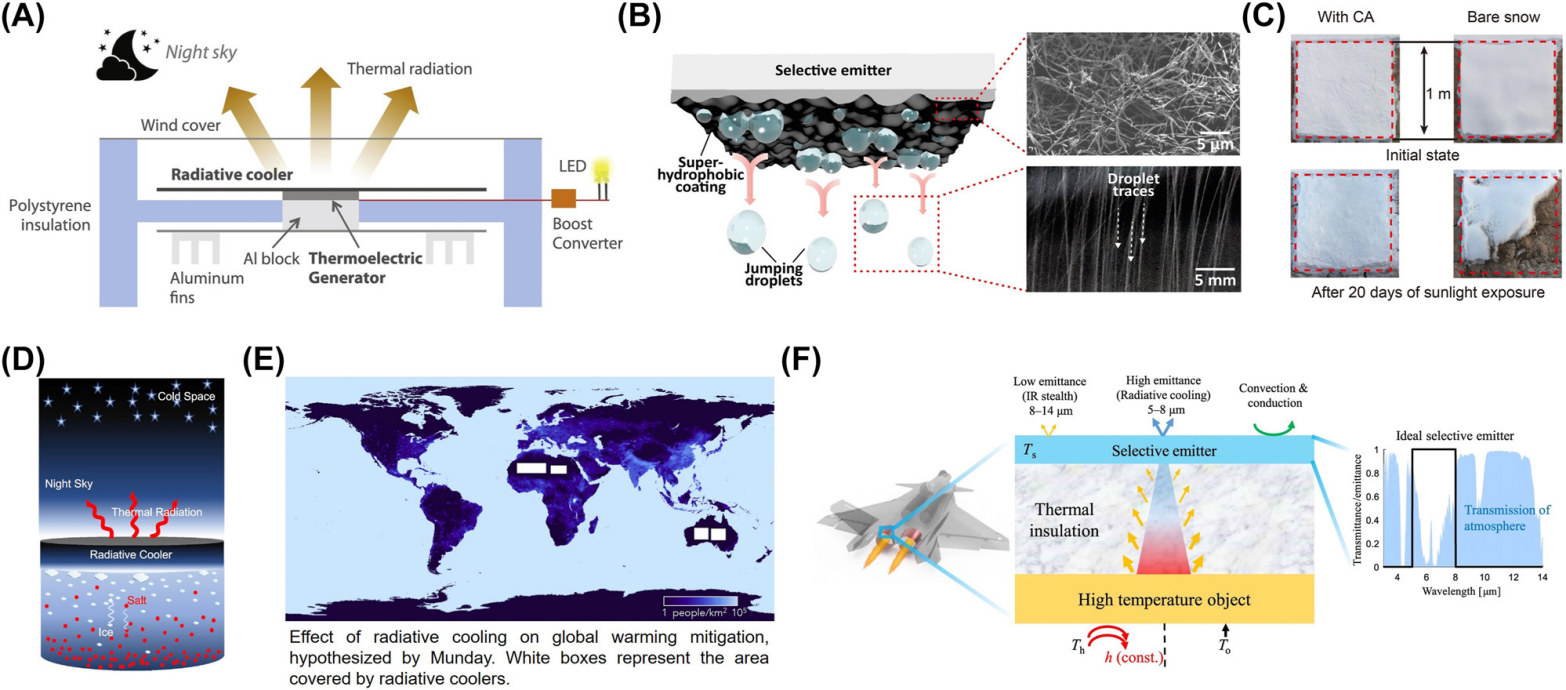
Potential and extension applications. (A) Nonstop work of thermoelectric generator by using the temperature difference between radiative cooler and the ambient [28]. (B) Water droplets condensate on and then self-remove from the surface of superhydrophobic radiative cooler [30]. (C) A comparison of snow layer with (left) and without (right) protection of radiative cooler after long-term sunlight exposure [97]. (D) Conceptual framework of the radiative cooling freezing desalination process [98]. (E) A bold hypothesis to mitigate global warming by setting radiative coolers on almost no-man’s desert regions [99]. (F) Schematic illustration of high-temperature infrared camouflage combining a wavelength-selective emitter and a thermal insulator. It is an example for reverse utilization of transparent atmospheric window [100]. Panels adapted with permission from Elsevier Limited, AAAS, and Springer Nature Limited.

Daytime subambient radiative cooler could make an all-day temperature difference with the ambient. In several studies, the cooler is integrated with a thermoelectric generator, where the cooler serves as the cold side and the surrounding air is the hot side. In this situation, thermoelectric devices could continue to produce electricity based on the Seebeck effect (Figure 9A). The measured output power exceeds 100 mW/m^2^ at night [101], large enough to power some electronics, such as light-emitting diodes (LEDs) and sensors [28]. The researchers even predict that the output power could be over 2 W/m^2^ by combination of spectro-angular-selective cooler and optimized thermoelectric system, almost two orders of magnitude higher than the reported experimental results [41]. In general, this design paves a path to sustainable and add-up power generation throughout the day and into the night.

#### 6.4.2 Water harvesting

Dew is water in the form of droplets, which forms on a cold surface with temperature below the dew point. Radiative cooler has a cold and even subambient surface temperature, which could efficiently improve the possibility and enhance the yield of dew. The water is harvested from the atmosphere without any external energy consumption (Figure 9B). It has been reported that the measured water-harvesting mass flux is over 50 g/(m^2^ h) [30], [31], and the ability to harvest water could be further enhanced by combining with absorption effect [102], [103].

#### 6.4.3 Ice storage

When the ambient temperature drops further, the surface temperature of the cooler will be below the freezing point of water. Dew on the surface takes the form of ice, and the existing ice is protected to prevent melting under the solar radiation. This idea could be considered as the extension of water harvesting and has been successfully demonstrated in experiment (Figure 9C) [97].

#### 6.4.4 Desalination

Different from water harvesting from the atmosphere, fresh water in this strategy is drawn from salt water. The ice layer forms at the bottom of a sky-facing radiative cooler with the temperature below the freezing point of water, while the remaining brine with higher salinity sinks because of its higher density. The separated ice is re-melted to obtain fresh water (Figure 9D) [98]. The researchers point out that this radiative cooling-driven freezing desalination could be further combined with solar heating-driven evaporating desalination, to realize year-round and all-weather passive thermal desalination in the future.

#### 6.4.5 Global warming mitigation

The report states that the earth is solar-absorbing ∼1 W/m^2^ more than it is thermal-radiating currently, which leads to an overall warming of the climate [104]. Some researchers make a global strategy, which is an extremely bold imagination. If only 1 %–2 % of the earth were covered by the existing radiative cooler with daytime net cooling power of ∼100 W/m^2^, the total heat fluxes into and away from the earth would be balanced and global warming would end (Figure 9E) [99], [105]. Other researchers put forward their own views. Large-scale radiative coolers could lead to uneven temperature variations that may result in further long-term climatic and environmental changes, while the fluctuated weather and climate also influence the cooling effect of radiative coolers in turn. In addition, from a commercial perspective, technology as mature as solar panels still don’t reach that level of cover after decades of development, it is likely impossible that this nascent technology could do so in time [99]. Therefore, imaginations of using radiative coolers for geoengineering to mitigate global warming seem further off. Nowadays, this imagination is still left on the theoretical shelf.

#### 6.4.6 High-temperature infrared camouflage

There are two main infrared transparent atmospheric windows, which locate on 3–5 μm and 8–13 μm, respectively. Infrared detector could be easy to monitor high-temperature objects through these two windows. As the temperature of a blackbody increases, the thermal radiation increases sharply in intensity and there its maximum shifts to shorter wavelengths. If the surface emissivity of a spectral-selectivity object would be modulated to be low in the atmospheric window while high in the other wavelength range, this object could have both infrared camouflage and radiative cooling (Figure 9F) [100], [106]. This concept has been demonstrated based on multi-layered materials and extends the application of radiative cooling technology at high temperature.

### 6.5 Other challenges for serving practice

There are some rest of common problems that should be faced when promoting radiative cooling to the practical applications. These problems cover producing, using, recycling, and other processes. If the studies are expected to face the real applications, the listed challenges as following have to be considered at the beginning of design.

#### 6.5.1 Large-sized production

**Figure 10:**
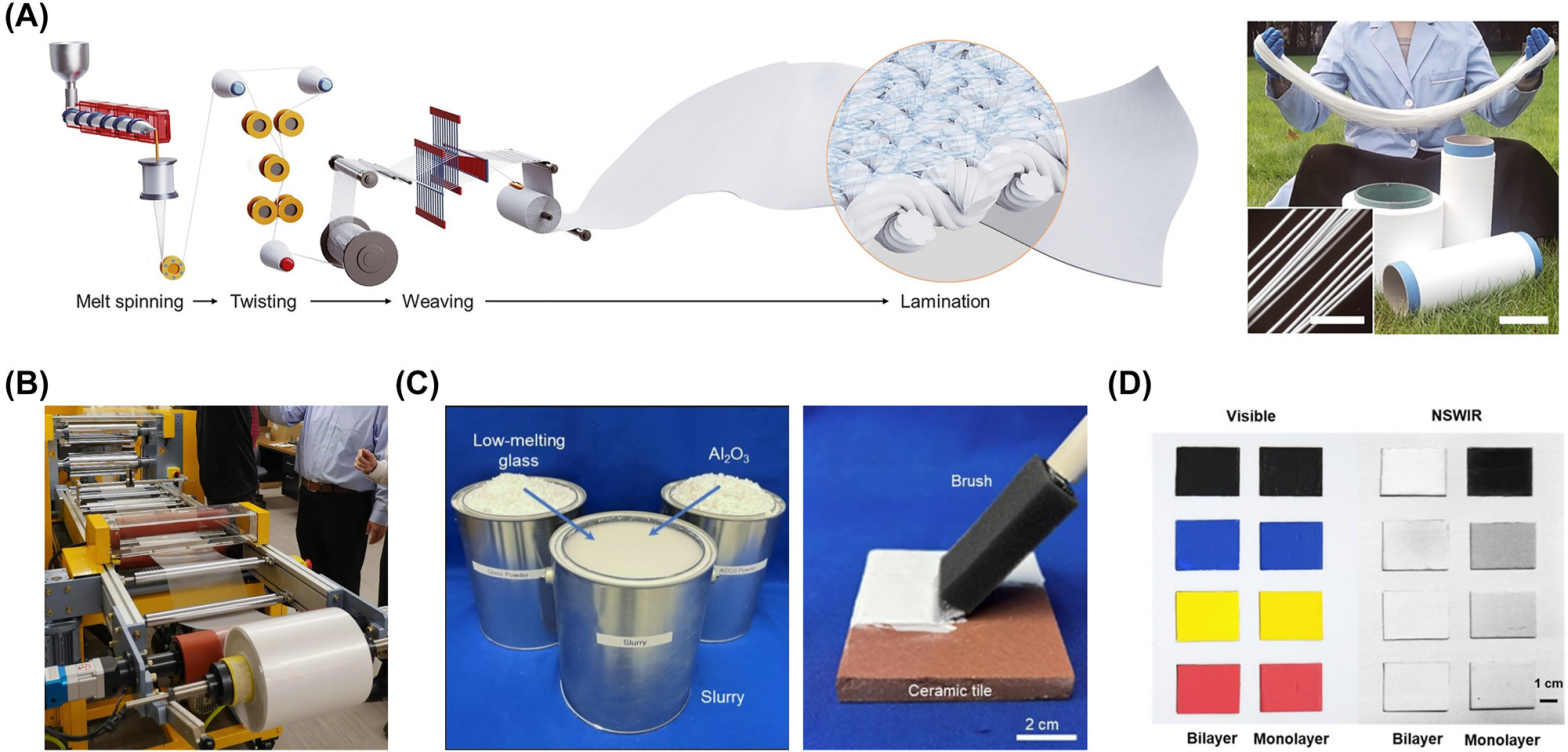
Parts of responses to challenges for serving practice. (A) Schematic illustration of assembly line for large-scale preparation of radiative cooling fabrics (left). Radiative cooling fibers with randomly distributed particle structure are prepared by melting spinning (right) [19]. (B) Radiative cooling film with randomly distributed particle structure is large-scale produced by roll-to-roll manufacturing method [18]. The picture is from the website of Colorado University [107]. (C) Radiative cooling paints are composed of low-cost raw materials (left) and could be applied on the surface of the object by brush painting, air spray coating, or blade coating, same as the commercial (right) [22]. (D) Colorful radiative cooling paints show the same appearance as the commercial, but an opposite near-infrared emission [108]. Panels adapted with permission from AAAS.

In the laboratory, the sample is often small sized, but enough to character all its properties. On the contrary, it is necessary that the products are large sized to meet the demands in the real applications. Limited by rigorous structure and preparation methods, some designs are impossible for large-sized products or their uniformity declines with the increasing size. This problem often occurs at the materials with layered or arrayed photonic structures. Spray-coating [20], solution-processed coating (Figure 10C) [22], [23], roll-to-roll manufacturing (Figure 10B) [18], [48], [109], [110], spinning (Figure 10A) [19], and other methods could make it possible to large-size production of materials with porous structure or randomly distributed particle structure.

#### 6.5.2 Large-scale production

Large-sized production is the demand on the size of products, while the large-scale is on the number of products during the producing process. On the one hand, large-scale production will efficiently cut the cost. On the other hand, it is considered that a large number of radiative coolers are used together to cause cluster effect in different potential applications. This is another challenge to the materials requiring stringent and nanometer-precision fabrication. Among radiative cooling materials with different photonic designs, one with porous structure or randomly distributed particle structure is easy to large-scale produce by economical roll-to-roll manufacturing (Figure 10B) [18], [48], [109], [110] or spinning (Figure 10A) [19] technology, which is almost embedded into the assembly line directly only in terms of preparation method. Recently, a meta-surface concept with periodically arranged three-dimensional (3D) trench-like structures is also demonstrated by roll-to-roll manufacturing technology [111]. This study opens a possible way to large-scale production of radiative cooling materials with layered or arrayed photonic structures.

#### 6.5.3 Colorful appearance

For the goal of daytime subambient radiative cooling, materials are designed to have a high solar reflection from the beginning. In doing so, they always exhibit a typically white or silver appearance. However, bright white or silver glare is an unfriendly light pollution in the real world, harmful to the human eyes. Meanwhile, monotonous color is undesirable for aesthetic or functional reasons. Some research groups have started paying attention to developing colorful radiative cooling materials (Figure 10D) [108], [112], [113]. Still, their colors are not as good as those of commercial paints.

#### 6.5.4 Durability

Specific requirements of durability on radiative cooling are that various physical and chemical shocks from the surrounding have no impact on its intrinsic spectral characteristics. The former includes scratching, striking, and pollution, and the latter is such as oxidation, ultraviolet aging, and so on. It means that the coolers have abrasive resistance, toughness, pollution resistance, and chemical inertness. In a word, intrinsic spectral characteristics should maintain unchanged during the working process. Nowadays, some experimental demonstrations give a series of solutions aiming to a kind or parts of influences, for example, hydrophobic property against dust pollution [20], [21], [23], [46] and selection of anti-ultraviolet materials or fluorination modification against ultraviolet aging [46], [114], [115].

#### 6.5.5 Recycling

The studies on radiative cooling mainly focus on the use but have less discussion on how to treat the waste radiative cooling materials. Recycling is a key component of modern waste reduction, which could be generally divided into physical recycling and chemical recycling. There seems to be a contradiction between required robustness and recycling of radiative cooling materials. That should be a matter of concern to plan an efficient recycling process. Several earlier attempts have been done based on solubility of polymer-based materials, typically cellulose [116]–[118]. Cellulose could be recycled and reused from its aqueous solution, but there seems to be a new problem – will rain or dew in nature destroy the spectral selectivity of cellulose-based radiative cooling materials, leading to degradation of cooling performance? In addition, recycling of other blends is still an unanswered question.

#### 6.5.6 Cost-effectiveness analysis

The analysis of cost-efficiency needs to be done continuously throughout the whole process, including designing, producing, using, recycling, except for certain specific applications that are not affordable sky-high cost likewise. The ultimate goal of radiative cooling is energy saving. Thus, the total energy consumption in the whole process must be less than energy saving in the working process (Figure 10C) [22], [46]. Besides these, such as utilization of space and time cost, everything that is referred to must be actuarially analyzed.

## 7 Conclusions

Radiative cooling comes from practice, is tested by practice, and will serve practice in turn. Radiative theory helps us build the framework to understand the thermodynamic process of radiative cooling in depth. The introduction of thermal photonics, laying at the interface between thermodynamics and photonics, causes an on-going upsurge of the studies on radiative cooling in the recent decade. The utilization of radiative cooling has been developed from local to global design. The researchers hope that radiative cooling, as a renewable cooling technology, could play a part in mitigation of global warming and further sustainable development. The studies on radiative cooling should be beyond just a few abstract papers. They will not only enrich the basic theory but also serve practice in the real world at the end.
